# Effects of Reactive Oxygen and Nitrogen Species on TrkA Expression and Signalling: Implications for proNGF in Aging and Alzheimer’s Disease

**DOI:** 10.3390/cells10081983

**Published:** 2021-08-04

**Authors:** Erika Kropf, Margaret Fahnestock

**Affiliations:** 1 Graduate Program in Neuroscience, Faculty of Health Sciences, McMaster University, Hamilton, ON L8S 4K1, Canada; kropfe1@mcmaster.ca; 2 Department of Psychiatry and Behavioural Neurosciences, Faculty of Health Sciences, McMaster University, Hamilton, ON L8S 4K1, Canada

**Keywords:** oxidative stress, nitrative stress, neurotrophin, p75^NTR^, basal forebrain, retrograde transport

## Abstract

Nerve growth factor (NGF) and its precursor form, proNGF, are critical for neuronal survival and cognitive function. In the brain, proNGF is the only detectable form of NGF. Dysregulation of proNGF in the brain is implicated in age-related memory loss and Alzheimer’s disease (AD). AD is characterized by early and progressive degeneration of the basal forebrain, an area critical for learning, memory, and attention. Learning and memory deficits in AD are associated with loss of proNGF survival signalling and impaired retrograde transport of proNGF to the basal forebrain. ProNGF transport and signalling may be impaired by the increased reactive oxygen and nitrogen species (ROS/RNS) observed in the aged and AD brain. The current literature suggests that ROS/RNS nitrate proNGF and reduce the expression of the proNGF receptor tropomyosin-related kinase A (TrkA), disrupting its downstream survival signalling. ROS/RNS-induced reductions in TrkA expression reduce cell viability, as proNGF loses its neurotrophic function in the absence of TrkA and instead generates apoptotic signalling via the pan-neurotrophin receptor p75^NTR^. ROS/RNS also interfere with kinesin and dynein motor functions, causing transport deficits. ROS/RNS-induced deficits in microtubule motor function and TrkA expression and signalling may contribute to the vulnerability of the basal forebrain in AD. Antioxidant treatments may be beneficial in restoring proNGF signalling and axonal transport and reducing basal forebrain neurodegeneration and related deficits in cognitive function.

## 1. Nerve Growth Factor Receptors and Signalling

Mature nerve growth factor (NGF) is a 13.2 kD protein that is essential for cell survival, synaptic plasticity, neurite outgrowth, and differentiation [1,2]. NGF binds to the tyrosine kinase receptor, TrkA, to elicit its neurotrophic functions [1,3]. NGF-induced TrkA activation initiates three main signalling cascades, the phosphatidylinositol-3-kinase (PI3K)-Akt pathway, the Ras-mitogen activated protein kinase-extracellular signal regulated kinase (Ras-MAPK-ERK) pathway, and the phospholipase C-gamma (PLC-γ) pathway [4,5,6,7]. Activation of the PI3K-Akt pathway is required for NGF-induced cell survival, while both Ras and PLC-γ signalling contribute to NGF-induced neurite outgrowth [4,8,9]. In addition to activation of the ERK signalling cascade, NGF activates the p38 MAPK pathway downstream of Ras [10]. Both of these pathways induced by NGF contribute to activating phosphorylation of the transcription factor cAMP-response element binding protein (CREB) at serine-133 [10]. CREB regulates various genes associated with beneficial functions including neurogenesis, neuronal survival, synaptic plasticity, and cognitive function [11].

Mature NGF also binds with low affinity to the pan-neurotrophin receptor, p75^NTR^ [1]. In the absence of TrkA, NGF-induced activation of p75^NTR^ induces apoptosis by activating ceramide and c-Jun N-terminal kinase (JNK) signalling [1,7,12]. However, in the presence of TrkA, p75^NTR^ enhances the neurotrophic functions of NGF by increasing the affinity of NGF for TrkA, increasing NGF/TrkA internalization, and inducing signalling via Akt to increase cell viability [1,7,13]. Activation of TrkA inhibits the apoptotic signalling of p75^NTR^ via the PI3K-Akt and Ras-MAPK-ERK pathways, which inhibit apoptotic signalling factors and activate anti-apoptotic factors, respectively [7].

The NGF gene contains two different promoters, and the resulting transcripts can be alternatively spliced to produce two major and two minor mRNA sequences [14,15]. Translation of NGF from the two major transcripts results in preproNGF species of 34 kDa and 27 kDa [16]. Following removal of the signal peptide in the endoplasmic reticulum, 32 and 25 kDa proNGF species remain [14,17,18,19,20]. These species can be further processed by various proteases to produce the mature, 13.2 kDa form of NGF [21,22,23]. However, in the human, rat, and mouse brain, proNGF is the predominant species, while mature NGF is not detected [24]. ProNGF exists as a 64 kDa dimer that has similar biological activity to mature NGF, although with lower potency [25,26]. Several studies indicate that proNGF binds to TrkA, albeit more weakly than mature NGF, and elicits TrkA phosphorylation and activation of downstream signalling factors including MAPK, ERK1/2, and Akt [25,27,28,29]. There is extensive literature indicating that proNGF exhibits a similar neurotrophic function to mature NGF in its ability to promote cell survival and neurite outgrowth [25,27,28,29,30,31,32,33]. Several of these studies utilized cleavage-resistant proNGF mutants and performed experiments in the presence of protease inhibitors to confirm that the observed neurotrophic effects were induced by proNGF rather than its mature form [25,27,28,29]. In these studies, the lack of proNGF cleavage was also confirmed via Western blot, providing further confidence that proNGF is neurotrophic [25,27,28,29].

Despite extensive literature supporting the role of proNGF as a neurotrophic factor, other evidence implicates proNGF in apoptosis [21,34]. These contradictory results can be explained by differences in proNGF receptor expression [28]. In the absence of TrkA, proNGF activates apoptotic signalling through p75^NTR^, together with sortilin [21,28,29,34]. However, in the presence of TrkA, proNGF retains neurotrophic activity [25,26,27,28,29]. Therefore, receptor expression is critical in determining the cellular outcome of proNGF activity [28,29,35].

## 2. Basal Forebrain Cholinergic Neurons: Dependence on NGF and Implications in Alzheimer’s Disease

Alzheimer’s disease (AD), the most common form of dementia, is characterized by progressive learning and memory deficits and accumulations of amyloid-beta and hyperphosphorylated tau proteins [36]. One brain area that is particularly vulnerable to AD is the basal forebrain [37]. Degeneration of basal forebrain cholinergic neurons (BFCNs), which are critical for learning, memory, and attention, contributes to the cognitive decline seen in aging and AD [38,39,40,41,42]. BFCN degeneration is also a feature of Down’s syndrome (DS), a trisomy of chromosome 21 that includes the amyloid precursor protein gene and produces AD-like pathology [43,44].

BFCNs rely on NGF for their survival and function [35,45,46,47,48,49,50,51]. Early studies demonstrated the presence of NGF mRNA overlapping with cholinergic subfields within the horizontal limb and diagonal band of broca within the basal forebrain, suggesting that these neurons supplied their own NGF [52,53]. However, later studies revealed that this NGF was present exclusively within GABAergic, not cholinergic, neurons within these regions [54]. Early in vivo work indicated that fimbria–fornix transection results in BFCN cell death and reduced learning and memory functions, both of which can be rescued by the addition of NGF [51,55]. These observations, coupled with the high levels of NGF present within the cortical and hippocampal targets of BFCNs, suggest that these neurons are reliant on retrograde axonal transport for their supply of NGF [53].

Mature NGF, bound to its receptors on axon terminals, is retrogradely transported from synaptic terminals to cell bodies in signalling endosomes, which contain TrkA and its downstream signalling factors and activate downstream signalling pathways along the length of the axon and in the cell body [56,57,58,59,60]. Recent evidence indicates that proNGF is also retrogradely transported by neurons in both the central and peripheral nervous systems and is transported in signalling endosomes, similarly to mature NGF [61,62,63].

**Figure 1:**
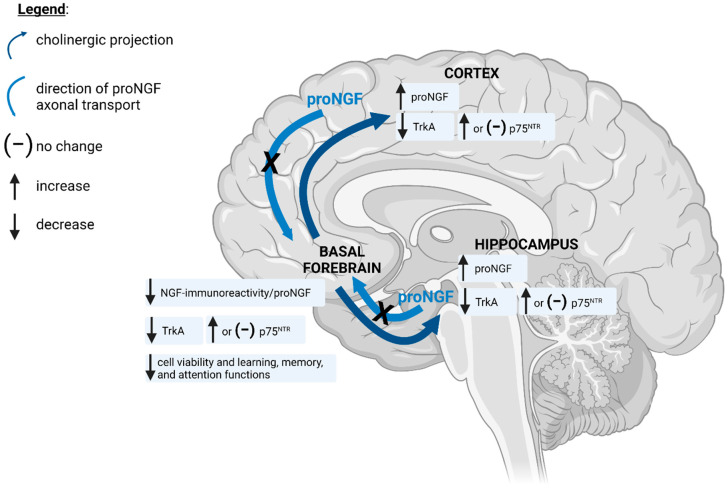
Alterations in proNGF distribution and receptor expression in aging and Alzheimer’s disease. Basal forebrain cholinergic neurons (BFCNs) send projections widely throughout the cortex and hippocampus. In the healthy brain, BFCNs receive neurotrophic support from these brain areas via retrograde transport of pro-nerve growth factor (proNGF). In aging and Alzheimer’s disease (AD), proNGF accumulates throughout the cortex and hippocampus, with accompanying decreases in proNGF/NGF-immunoreactive material in the basal forebrain. Decreased tropomyosin-related kinase A (TrkA) levels are observed in the basal forebrain, cortex, and hippocampus, with either increased or no change in levels of the pan-neurotrophin receptor, p75^NTR^. These alterations are associated with BFCN degeneration and loss of learning, memory, and attention in aging and AD. Diagram was created using BioRender.com (accessed on 4 August 2021).

The aged basal forebrain exhibits impairments in the retrograde transport of NGF as well as in general axonal transport [64,65,66]. In AD and DS, NGF immunoreactivity accumulates throughout the cortex and hippocampus and is reduced in the basal forebrain, indicating a deficit in its retrograde transport (Figure 1) [59,67,68,69,70,71]. However, these studies did not differentiate mature NGF from its precursor form, as the molecular weight of the detected proteins was not analyzed. The antibodies used for NGF detection via EIA, ELISA, dot blot, and immunohistochemistry in these studies, directed against mature NGF, also recognize proNGF. More recent studies demonstrate that the previously reported accumulations of mature NGF are actually proNGF rather than its mature form [24,63,72,73]. Similar proNGF accumulations are observed in the cortex of patients with DS, AD, and mild cognitive impairment, as well as in DS and AD rodent models [72,73,74,75]. Processing of proNGF to its mature form may be impaired in DS and AD, which may contribute to proNGF accumulation [75,76,77]. However, the lack of detectable NGF in healthy or diseased brain, the regional specificity of proNGF accumulation and the decreased immunoreactivity in the basal forebrain are highly suggestive of impaired retrograde transport deficits. No differences in the expression of NGF mRNA are observed in the aged or AD brain despite observed increases in NGF-immunoreactive protein in the hippocampus and cortex and decreases in the basal forebrain, supporting the role of retrograde transport [69,78,79]. Furthermore, deficits in proNGF retrograde axonal transport have been reported in BFCNs aged in vitro [35,63]. Similar reductions in the retrograde transport of ^125^I-NGF and cholinergic deficits are observed in DS mice and aged rats [59,64]. Thus, retrograde transport of proNGF in BFCNs is impaired in cellular models of aging and in DS and AD animal models and likely also in DS and AD.

The accumulation of proNGF in the cortex and hippocampus reduces BFCN survival and function. In mild cognitive impairment and early stages of AD, cortical proNGF accumulation is inversely correlated with cognitive scores [72,80]. The accumulation of proNGF in the rat cortex, mimicking the accumulation seen in AD, causes degeneration of the BFCNs that innervate this area, indicated by decreased BFCN soma size and reduced cortical cholinergic innervation and synapses as well as reduced learning and memory [80,81].

Reductions in TrkA expression and signalling are also observed in the AD brain, with either no observed differences or an elevation in p75^NTR^ expression (Figure 1) [64,82,83,84,85,86,87,88]. Similar reductions in TrkA and no change in p75^NTR^ expression are found in animal and cellular models of aging, DS, and AD, and are related to a loss of cholinergic markers and deficits in cognitive function [63,64,89]. The etiology of deficits in proNGF retrograde transport and TrkA expression and signalling in aging, AD, and DS basal forebrain has yet to be confirmed. Emerging literature indicates that oxidative and nitrative stress may be contributing factors. Supporting evidence is discussed in the remainder of this review.

## 3. Oxidative and Nitrative Stress in Neurodegenerative Disease

Highly reactive molecules with unpaired electrons in their outer valence, called free radicals, are a natural by-product of cellular energy metabolism [90]. Free radicals containing oxygen are classified as reactive oxygen species, while those that also contain nitrogen are considered reactive nitrogen species. Reactive oxygen and nitrogen species (ROS/RNS) are commonly produced in mitochondria and are released from microglia during inflammatory responses [90]. ROS/RNS add oxidative and nitrative modifications to cellular components that result in cell damage and death when they are present in high amounts [91]. Under normal conditions, ROS/RNS are quenched by antioxidant systems, preventing them from causing cellular and molecular damage [92].

Oxidative and nitrative stress result when there is an imbalance between ROS/RNS production and ROS/RNS removal by antioxidants [90,92]. In aging and AD, mitochondrial deficits and overactivation of microglia, along with a concurrent decline in antioxidant systems, contribute to the overproduction of ROS/RNS [92,93,94,95]. The resulting oxidative and nitrative stress generates extensive cellular and molecular damage in the AD brain [93]. Increased markers of oxidative and nitrative damage, including lipid peroxidation, oxidation of macromolecules, protein carbonyls, and nitrotyrosine residues, are observed in human post-mortem brain tissue from AD patients [95,96,97,98,99,100]. Elevations in ROS/RNS occur prior to AD pathology in animal models, suggesting that the increases in oxidative and nitrative stress that occur in normal aging are further exacerbated in pathological aging such as AD [101]. Oxidative and nitrative modifications are present within tau neurofibrillary tangles and amyloid-beta plaques [102,103,104]. Several studies show that ROS stimulate amyloid-beta production and accumulation as well as tau phosphorylation and oligomerization, indicating that oxidative stress contributes to the generation of AD pathology [105]. Amyloid-beta accumulation also generates ROS, indicating that ROS may interact with AD pathology in a positive feedback loop [93].

## 4. ROS/RNS-Induced Deficits in BFCN Viability and Cognitive Function Are Associated with Nitration of proNGF and Reduced TrkA Expression and Signalling

The basal forebrain is especially vulnerable to ROS/RNS [106]. Extensive literature indicates that oxidative and nitrative damage and deficits in antioxidant systems are associated with the cholinergic dysfunction and cognitive decline seen in aging and AD [107,108,109,110,111,112,113,114]. Oxidative stress contributes to BFCN degeneration and the associated cognitive decline, and antioxidant treatment delays these pathologies [115]. RNS also produces nitration of proNGF in the cortex of aged and cognitively impaired rats [107]. Nitrated proNGF is the main NGF species detected in the AD brain and is correlated with a loss of cholinergic markers and impaired memory performance [107,116]. Nitrated proNGF accumulates in both the human AD cortex and in rodent models of AD [116]. Further investigation is required to elucidate the cause of such accumulation. Potential contributing factors include impaired retrograde transport of proNGF to the basal forebrain and deficits in proNGF maturation. Nitration reduces the ability of mature NGF to activate TrkA and may have a similar affect on proNGF due to the similarity between mature NGF and proNGF in TrkA binding and activation [27,116]. However, this must be tested for proNGF specifically, as proNGF and mature NGF exhibit differences in their TrkA binding affinity and potency of activation of signalling factors [25,27].

ROS/RNS-induced deficits in cognitive and cholinergic functions are also associated with decreased expression of TrkA [117]. Amyloid-beta, which increases oxidative stress in the brain, causes cholinergic dysfunction that is associated with reductions in TrkA expression and memory impairments [106,117]. Antioxidant treatments increase NGF-dependent activation of ERK and CREB and rescue TrkA expression and cognitive and cholinergic functions in rodent models of neurodegenerative disease [117,118]. Together, these findings indicate that the loss of cognitive function induced by oxidative and nitrative stress is associated with disruptions in TrkA expression and signalling.

Several studies indicate that mature NGF is protective against oxidative stress by stimulating antioxidant responses [119,120]. Conversely, deprivation of mature NGF increases ROS, possibly by decreasing mitochondrial function [121]. In PC12 cells, treatment with mature NGF increases cell survival via the PI3K pathway in response to oxidative and nitrative stress [122,123]. In SH-SY5Y cells treated with hydrogen peroxide, antioxidant treatment increases mature NGF protein levels and rescues cell survival via a mechanism dependent on TrkA activation and the activity of the downstream MAPK/ERK signalling pathway, implicating TrkA signalling in defense against oxidative damage [124]. Thus, NGF availability and signalling contribute to cellular protection against oxidative and nitrative insult. However, these effects have yet to be tested specifically for proNGF in the basal forebrain. Nevertheless, overall, these studies highlight the importance of preserving TrkA signalling in the basal forebrain to maintain neuronal protection against oxidative damage and ROS-associated cognitive decline.

## 5. Oxidative and Nitrative Stress Alter the Expression and Signalling of proNGF Receptors

Elevations in ROS may cause an imbalance in TrkA and p75^NTR^ receptors in the aging and AD basal forebrain, as several studies indicate that oxidative and nitrative stress alter the expression of these receptors. Similar to what is observed in the aging and AD brain, treatment with ethanol or amyloid-beta, both of which generate oxidative stress, decreases TrkA mRNA and protein expression in the basal forebrain and hippocampus [117,125,126,127]. TrkA immunoreactivity is also decreased in the basal forebrain of DS mice and in BFCNs aged in vitro, both of which exhibit elevations in ROS [63,115]. Reductions in TrkA mRNA and protein expression in vitro and in vivo can be rescued via antioxidant treatment, indicating a causal role of ROS in TrkA depletion [115,117,124,127,128]. Together, these studies indicate that oxidative stress likely contributes to the decreases in TrkA expression observed in the aging and AD brain. 

The reported effects of ROS/RNS on p75^NTR^ expression are inconsistent. Oxidative treatment via ethanol or monocrotophos does not affect p75^NTR^ protein expression in the rat basal forebrain or in neural stem cells [125,129]. These results are consistent with evidence indicating no differences in p75^NTR^ expression in the human AD basal forebrain, aged rodent basal forebrain, or rat BFCNs aged in vitro [63,64,82,83]. However, other studies indicate that the protein levels of p75^NTR^ are increased by oxidative and nitrative stress induced by hydrogen peroxide, amyloid-beta, peroxynitrite, monocrotophos, and chlorpyrifos in the mouse SN56 basal forebrain cell line, human SH-SY5Y cells and retina, and rat cortex and hippocampus [128,129,130,131]. These results are consistent with other literature indicating an elevation of p75^NTR^ in the human AD basal forebrain and entorhinal cortex [86,88]. Differing results may be due to differences in cell types, species, and brain areas studied, as well as variations in treatment conditions used to generate oxidative and nitrative stress.

Maintaining the balance of TrkA and p75^NTR^ levels at the axon terminals is critical for regulation of proNGF survival vs. apoptotic signalling [28,29]. ProNGF is neurotrophic in the presence of TrkA but exerts apoptotic effects via p75^NTR^ when TrkA is absent [28,29,35]. Therefore, ROS-induced reductions in axonal TrkA expression, with concurrent maintenance or elevation in p75^NTR^ expression, are expected to decrease proNGF-TrkA survival signalling while increasing proNGF-p75^NTR^ apoptotic signalling. In fact, oxidative and nitrative stress increase the activation of apoptotic signalling factors downstream of p75^NTR^, such as caspase-3, Bax, JNK, and nuclear factor kappa light chain enhancer of activated B cells (NF-κB), in a variety of cell types including neurons, astrocytes, and PC12 cells [123,129,132,133]. Conversely, oxidative stress prevents TrkA phosphorylation and activation of downstream signalling factors such as Akt, ERK1/2, and CREB in neural stem cells, PC12 cells, and astrocytes [129,132,134]. Further, SH-SY5Y cells expressing a presenilin-1 (PS1) mutation causing increased amyloid-beta production, which generates oxidative stress, exhibit impaired NGF-stimulated activation of TrkA and MAPK and mislocalization of TrkA from the membrane to the cytoplasm and nucleus [135]. NGF-induced TrkA phosphorylation and activation of MAPK are also reduced by nitrative stress caused by peroxynitrite in PC12 cells [136]. In the diabetic retina, peroxynitrite decreases TrkA phosphorylation at tyrosine 490 (Y490), the site required for stimulation of the PI3K-Akt survival pathway. This prevents Akt activation, implicating nitrative stress in decreased TrkA signalling and associated reductions in cell viability [130]. Decreased Akt activity is also associated with the TrkA nitration observed in retinal ganglion neurons [130]. TrkA nitration has yet to be assayed in the degenerating basal forebrain. Together, these results highlight that oxidative and nitrative stress decrease TrkA signalling while increasing that of p75^NTR^, leading to neurodegeneration and cell death.

On the other hand, some studies indicate that TrkA signalling is maintained or elevated in response to oxidative and nitrative stress. Nutrient deprivation-induced ROS elevation activates TrkA by decreasing membrane cholesterol in PC12 cells, while hydrogen peroxide and peroxynitrite induce activation of p38 in PC12 cells and diabetic retina [130,137,138]. These findings may be due to differences in reagents used to generate oxidative and nitrative stress as well as differences in the extent and duration of the stress [133]. The effects of ROS/RNS on TrkA and p75^NTR^ signalling in the basal forebrain require further investigation.

Although many of these studies use mature NGF to stimulate TrkA and to investigate the relationship between ROS/RNS and downstream signalling, it is likely that these mechanisms are conserved between proNGF and mature NGF. ProNGF is similar to mature NGF in its ability to activate TrkA, induce pro-survival signalling and neurite outgrowth, and be retrogradely transported [25,27,28,29,30,31,32,33,61,62]. However, because the two NGF species exhibit minor differences in their affinity for TrkA binding and activation, the effects of oxidative and nitrative stress on proNGF signalling must be tested [27].

## 6. Oxidative and Nitrative Stress Interfere with the Axonal Transport Machinery Required for Retrograde Transport of proNGF

Cytoplasmic dynein is the molecular motor required for the retrograde transport of proNGF and activated Trks [139,140]. The motor domain of dynein, which allows it to interact with and move along the microtubules, is contained within its heavy chain subunit [141]. The dynein heavy chain forms a complex with intermediate and light chains, which regulate cargo binding and heavy chain motor activity [141]. Sequencing studies indicate that dynein intermediate and light chains in flagellar dynein, which have sequence homology to that of cytoplasmic dynein, contain redox-sensitive regions that may be involved in alteration of dynein motor activity [141,142,143]. These regions contain dithiol groups that are highly sensitive to oxidative changes. Oxidation of these residues alters dynein ATPase activity, suggesting that oxidative stress may impair dynein-dependent transport by modification of the dithiol-containing regions [141,142,143]. Interestingly, dynein intermediate and light chains contain the same active site as thioredoxin, an enzyme involved in redox signalling [142,143]. The active site of thioredoxin can be oxidized to repress its activity, providing further evidence that dynein motor function may be inhibited by oxidative stress [144].

Oxidative and nitrative stress may also affect dynein-dependent axonal transport by altering the expression of key components of the dynein motor [145]. Nitrated tubulin, which is elevated in conditions of nitrative stress, decreases the dynein heavy chain protein and alters its distribution, indicating an impaired association between dynein and microtubules [146]. Similarly, rotenone, a chemical that elevates ROS and generates oxidative damage, decreases the protein expression of dynein heavy chain in cell cultures obtained from the hippocampus, locus coeruleus, and substantia nigra [145,147,148]. Similar decreases are also seen in aged rats following in vivo treatment with rotenone [145]. Protein expression of dynactin, a dynein-associated protein, is also decreased in the hippocampus by rotenone treatment [145]. Interestingly, in the locus coeruleus and substantia nigra, dynactin protein levels increase following in vivo rotenone treatment, which may be a compensatory response to the reduction in dynein protein levels [145]. The expression of kinesin, the motor protein required for anterograde transport, is also decreased by rotenone, and this decrease is associated with impairments in mitochondrial axonal transport [149].

Another mechanism by which oxidative and nitrative stress may disrupt axonal transport is by decreasing microtubule stability [150]. Peroxynitrite-induced tau nitration decreases the affinity of tau for microtubules and increases its aggregation [150]. Cells containing nitrated tau have altered morphology and neurite retraction, evidence of microtubule destabilization and degradation [150]. Similarly, oxidative stress induced by hydrogen peroxide causes axonal degeneration [151]. Because microtubules are critical for axonal transport, their degeneration caused by oxidative and nitrative stress may cause reductions in retrograde axonal transport [152]. Both tau nitration and microtubule degeneration are observed in the AD brain, suggesting that these pathologies may contribute to deficits in the retrograde transport of proNGF in AD [72,73,150,153].

Nitrative stress decreases axonal transport mediated by KIF1A, the kinesin motor responsible for the anterograde transport of TrkA [154,155,156]. Anterograde transport of TrkA is required for its expression at the axon terminals and subsequent retrograde transport of TrkA–neurotrophin receptor complexes [139,157]. When anterograde transport of TrkA is impaired by interfering with KIF1A, retrograde transport of TrkA is also disrupted [156]. Furthermore, mutations in presenilin-1 (PS1), which are linked to amyloid-beta accumulation and the generation of oxidative stress, cause the accumulation of TrkA in the cytosol and nucleus rather than expression at the cell surface [135]. These studies highlight that ROS/RNS may disrupt the kinesin-dependent axonal transport of TrkA.

## 7. Conclusions

**Figure 2:**
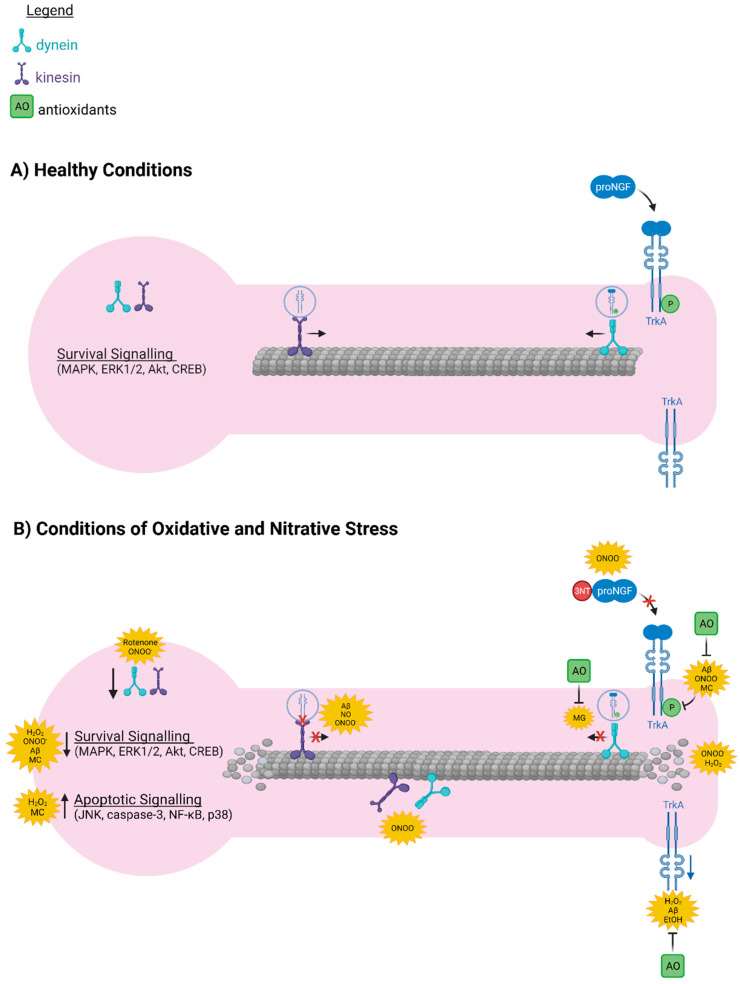
Oxidative and nitrative stress interfere with mechanisms of pro-nerve growth factor (proNGF) axonal transport. (**A**) Under healthy conditions, tropomyosin-related kinase A (TrkA) is expressed at axon terminals. Binding of proNGF induces TrkA autophosphorylation, internalization, and dynein-dependent retrograde transport. These events activate pro-survival signalling factors in the axon and at the cell body to maintain neuronal health. Kinesin motors transport somal TrkA receptors anterogradely to replenish and maintain axonal expression of TrkA. Kinesin and dynein molecular motors are expressed in the neuron and efficiently bind to microtubules to facilitate axonal transport of TrkA. (**B**) In conditions of oxidative and nitrative stress, such as in aging, Down’s syndrome (DS), and Alzheimer’s disease (AD), reactive oxygen and nitrogen species (ROS/RNS) decrease both protein and mRNA expression of TrkA. When nitrated, NGF is less efficient at activating TrkA and its downstream signalling factors, including mitogen-activated protein kinase (MAPK), phosphatidylinositol-3-kinase (PI3K), Akt, extracellular signal-regulated kinase 1/2 (ERK1/2), and cAMP response element-binding protein (CREB). ROS have the opposite effect on activation of signalling factors downstream of the pan-neurotrophin receptor, p75^NTR^, including c-Jun N-terminal kinase (JNK), nuclear factor kappa B (NF-κB), caspase-3, and Bcl-2-associated X protein (Bax), as all of these are increased in conditions of oxidative and nitrative stress. ROS/RNS decrease protein expression of kinesin and dynein molecular motors and interfere with their interaction with microtubules. ROS/RNS also suppress dynein motor activity and interrupt the interaction between kinesin and its cargo. Finally, ROS/RNS cause axonal degeneration and decrease the affinity of tau for microtubules, leading to microtubule instability and further disruption of axonal transport. Antioxidant treatments are effective in restoring TrkA expression, activation, and retrograde transport. The contributing ROS/RNS generators include amyloid-beta (Aβ), hydrogen peroxide (H_2_O_2_), ethanol (EtOH), monocrotophos (MC), methylglyoxal (MG), and peroxynitrite (ONOO^−^). Diagram was created using BioRender.com. 3NT: 3-nitrotyrosine. Up arrow represents increase, down arrow represents decrease.

Oxidative and nitrative stress produce multiple modifications that may impair the retrograde transport and signalling of proNGF by a variety of mechanisms (Figure 2A,B). The resulting reduction in proNGF in the basal forebrain is detrimental to BFCN survival and cognitive function. Which of these mechanisms are most relevant to the basal forebrain and its susceptibility to oxidative insult, neurodegeneration, and the associated cognitive decline in AD is an important area for future investigation.

Oxidative and nitrative stress decrease TrkA expression while either maintaining or elevating p75^NTR^ expression in the basal forebrain. The resulting receptor imbalance is accompanied by decreased survival signalling via TrkA and increased apoptotic signalling via p75^NTR^. Nitration of proNGF, altered function of dynein and kinesin molecular motors, and decreased microtubule stability may also contribute to these deficits. The effects of ROS/RNS-induced deficits in TrkA signalling on cell viability and cognitive function can be rescued with antioxidant treatment. Therapeutic interventions targeting elevations in oxidative and nitrative stress may therefore be beneficial in rescuing the retrograde transport of proNGF in the basal forebrain in aging and AD and subsequently improving BFCN survival and associated cognitive function.
